# Prices, availability and affordability of medicines in Rwanda

**DOI:** 10.1371/journal.pone.0236411

**Published:** 2020-08-03

**Authors:** Thomas Bizimana, Pierre Claver Kayumba, Lutz Heide

**Affiliations:** 1 Department of Pharmacy, School of Medicine and Pharmacy, College of Medicine and Health Sciences (CMHS), University of Rwanda, Kigali, Rwanda; 2 East African Community Regional Centre of Excellence for Vaccines, Immunizations and Health Supply Chain Management (EAC RCE-VIHSCM), University of Rwanda, Kigali, Rwanda; 3 Pharmaceutical Institute, Eberhard Karls University Tübingen, Tübingen, Germany; UKZN, SOUTH AFRICA

## Abstract

**Background:**

Access to affordable and good quality medicines is a key to meeting Sustainable Development Goal No. 3 by the year 2030. Prices, availability and affordability of essential medicines have been studied in many developing countries, but no such information has been published about Rwanda yet. This study aimed at providing data on prices, availability and affordability of medicines in different health facilities of Rwanda.

**Methods:**

A survey was carried out on availability, prices and affordability of 18 medicines in Kigali City and five districts of Rwanda. 44 health facilities were surveyed, including public and faith-based hospitals, public and faith-based health centers and private pharmacies. The standardized methodology developed by WHO and Health Action International (HAI) was used to collect and analyze the data.

**Findings:**

Prices for generic medicines in public and faith-based health facilities were remarkably low, with median price ratios (MPRs) of 1.0 in comparison to the international procurement prices published by Management Sciences for Health. In private pharmacies, prices were twice as high (MPR = 1.99 for generics). Availability of medicines fell short of the of 80% target set by WHO, but was better than reported from many other developing countries. Availability of medicines was highest in the private sector (71.3%) and slightly lower in the faith-based (62.8%) and public (59.6%) sectors. The government procurement agency was found to work efficiently, achieving prices 30% below the international procurement price given in the International Medical Product Price Guide. Affordability of medicines was better in the public and faith-based sectors than in the private sector.

**Conclusion:**

In Rwanda, medicines are affordable but poorly available in both the public and the faith-based sectors. Further improvements of the availability of medicines in the public and the faith-based health facilities represent the most important key to increase accessibility and affordability of medicines in Rwanda.

## Introduction

Access to quality essential medicines is part of the Sustainable Development Goal (SDG) No. 3 of the United Nations (UN) and a key to achieving Universal Health Coverage (UHC) by 2030 [1,2]. It is one of the five crucial policy areas identified by the “Lancet’s Commission on Essential Medicines Policies” in 2016 [3]. To achieve sustainable access to essential medicines for all people, affordability of essential medicines should be ensured especially in low and middle-income countries. The Lancet’s commission estimated that between US$13 and US$25 per capita (total US$ 77 to US$152 billion) is required annually to finance a basic package of 201 essential medicines (378 dosage forms) in all Low- and Middle-Income Countries (LMICs) [3]. In its “Global Action Plan for the Prevention and Control of Non-Communicable Diseases 2013–2020”, the 66^th^ World Health Assembly has formulated the voluntary global target: an 80% availability of the affordable basic technologies and essential medicines, including generics, required to treat major non-communicable diseases in both public and private facilities [4]. The “UN Commission on Life-Saving Commodities for Women and Children” estimated that improved access to and proper use of 13 life-saving commodities (for reproductive, maternal, newborn and child health) in 50 of the world’s poorest countries would prevent more than 6 million deaths of women and children in five years [5].

In 2003, the first edition of a standardized method for the measurement of medicine availability, prices and affordability was published by World Health Organization (WHO) in collaboration with Health Action International (HAI). The second edition of this methodology was published in 2008, and the global core list of included medicines was updated in May 2016 [6]. Surveys have been conducted using this methodology in many LMICs, frequently reporting low availability, high prices and correspondingly poor affordability of essential medicines [7].

With all these efforts at international and national levels, so far in Rwanda only a single, small study has been published on medicine availability. It investigated stock-out times of 10 essential medicines in 15 rural health centers in a single district of Rwanda in the year 2013, and reported that on average these medicines were out of stock for 2.7 months; quinine tablets were out of stock even for 10.5 months [8]. Beyond that report, no further results have been published about availability, prices and affordability of medicines in Rwanda. Therefore, in the present study we investigated availability, prices and affordability of 18 important medicines in public, private and faith-based health facilities in Kigali city and in five further districts of Rwanda, using the methodology published by WHO and HAI [6]. The results provide the first comprehensive picture of medicine availability and prices in Rwanda, and may help to identify the priorities for a further improvement of access to medicines in this country.

## Methods

### Ethical approval

This study was authorized by the Ministry of Health (Approval Notice No. 20/1361/DGPHFIS/2018) after ethical approval by the Institutional Review Board of the College of Medicine and Health Sciences of the University of Rwanda (Approval Notice No. 026/CMHS IRB/2018).

### Medicines included

Data were collected on prices, availability and affordability of 18 medicines (Table 1). 13 of these medicines are included in the WHO and HAI global core list [6]. Five supplementary medicines used in maternal and child health, especially in the management of post-partum hemorrhage, were also surveyed as this is a priority in Rwandan health policies. Of the 18 survey medicines, one (tranexamic acid injection 100mg/ml) was not included in the 2015 Rwanda Essential Medicines List (REML) [9,10], but has recently been recommended by WHO for post-partum hemorrhage (PPH) in addition to standard care [11]. Originator brands registered in Rwanda were identified using the list of medicines authorized in Rwanda which is published by the Ministry of Health [12]. According to this list, originator brands were registered in Rwanda for only eight of the 18 medicines included in this study (Table 1).

**Table 1:** Medicines included into this survey.

Survey medicines		Included in WHO/HAI Global Core List	Originator brand registered in Rwanda	Availability in the health system according to the 2015 REML		Daily dose [units or ml]	Treatment duration [days]	Number of units or ml for one course of treatment
				District hospital	Health center			
**Antibiotics**								
1	Amoxicillin capsule 500mg	Yes	-	**+**	**+**	**3**	**7**	**21**
2	Ceftriaxone injection 1g	Yes	-	**+**		**1**	**1**	**1**
3	Ciprofloxacin tablet 500mg	Yes	-	**+**	**+**	**2**	**7**	**14**
4	Co-trimoxazole suspension 8+40mg/ml	Yes	Yes	**+**	**+**	**10 ml**	**7**	**70 ml**
5	Metronidazole tablet 250mg	-	Yes	**+**	**+**	**6**	**7**	**42**
**Medicines against Non-Communicable Diseases (NCDs)**								
6	Amitriptyline tablet 25mg	Yes	-	**+**	**+**	**3**	**30**	**90**
7	Captopril tablet 25mg	Yes	-	**+**	**+**	**2**	**30**	**60**
8	Diazepam tablet 5mg	Yes	Yes	**+**	**+**	**1**	**7**	**7**
9	Diclofenac tablet 50mg	Yes	-	**+**	**+**	**2**	**30**	**60**
10	Metformin tablet 500mg	Yes	Yes	**+**	**+**	**3**	**30**	**90**
11	Omeprazole capsule 20mg	Yes	-	**+**		**1**	**30**	**30**
12	Salbutamol inhaler 100mcg/dose	Yes	Yes	**+**	**+**	**As needed**	**As needed**	**200 doses**
13	Simvastatin tablet 20mg	Yes	Yes	**+**		**1**	**30**	**30**
**Medicines for Maternal & Child Health**								
14	Levonorgestrel tablet 1.5mg	-	Yes	**+**	**+**	**1**	**1**	**1**
15	Misoprostol tablet 200mcg	-	Yes	**+**		**4**	**1**	**4**
16	Oxytocin injection 10IU/ml	-	-	**+**	**+**	**1 ml**	**1**	**1 ml**
17	Paracetamol suspension 24mg/ml	Yes	-	**+**	**+**	**15 ml**	**3**	**45 ml**
18	Tranexamic acid injection 100mg/ml	-	-	**Not included in the 2015 REML**		**5 ml**	**1**	**5 ml**

The Global Core List of survey medicines has been published by WHO and HAI [6] and updated in 2016 [7].

### Districts and regions surveyed

Six survey regions (administrative districts) were deliberately chosen with the purpose to represent the four provinces and the capital city of Rwanda: Karongi district in the Western province, Muhanga and Kamonyi districts in the Southern province, Musanze district in the Northern province, Bugesera district in the Eastern province, and Kigali city.

### Sectors surveyed

Availability of medicines, and prices which patients had to pay for these medicines, were recorded in health facilities/pharmacies in the public sector (outpatient only), in the private sector and in the faith-based sector. Furthermore, public procurement prices, i.e. prices payed by the “Rwanda Biomedical Center/ Medical Production, Procurement and Distribution Division (MPDD)” in its medicine purchases for the government health system, were collected from the central medical store, in accordance with the WHO/HAI manual.

### Health facilities surveyed

For each or the six survey regions, a list of all active health facilities was obtained from Ministry of Health files, and then organized into five categories: district hospitals, public health centers, faith-based health centers, private clinics and private pharmacies. For each survey region, two health facilities from each category were selected randomly, using the RND function of Microsoft Excel, provided two or more facilities were available. If there was only a single facility of the category in the region, that facility was selected.

Initially 57 health facilities were selected: 9 district hospitals, 12 public health centers, 12 faith-based health centers, 12 private clinics and 12 private pharmacies. One of the selected district hospitals had to be excluded from the study because it was found to be a specialized orthopedic hospital which was not stocking the surveyed medicines. Furthermore, according to Ministry of Health regulations [13], the selected private clinics were only authorized to keep emergency medications, not the survey medicines. Medicines prescribed in private clinics are dispensed from private pharmacies. However, in this study, ceftriaxone was found in 3 of the 12 private clinics, and oxytocin in one private clinic. Other private clinics may stock the survey medicines, but did not share this information with the investigator (as stocking these medicines may constitute a violation of the Ministry of Health rules). It was decided to exclude all 12 private clinics from data analysis. Therefore, a total of 44 health facilities were included in the data analysis (Table 2).

**Table 2:** Districts and health facilities from which data were collected for this study.

District	Public facilities		Faith-based facilities		Private facilities	Total
	Hospital	Health Centre	Hospital	Health Centre	Pharmacies	
Kigali City	2	2	0	2	2	8
Muhanga	0	2	1	2	2	7
Musanze	1	2	0	2	2	7
Karongi	0	2	2	2	2	8
Bugesera	0	2	1	2	2	7
Kamonyi	0	2	1	2	2	7
Sub-total	3	12	5	12	12	44
	15		17		12	

### Data collection

Data were collected from February-April 2019, entered into the automated Microsoft Excel workbook developed by WHO and HAI [6] and double-checked by the investigator to ensure accuracy of data. Following the WHO and HAI methodology [6], for each medicine data were collected on the originator brand (if registered in the country) and their generic equivalent. If several generic medicines were found in an outlet for a survey medicine, price data were recorded for the generic with the lowest unit price.

### Data analysis

Summary results such as percent availability, median price ratios and cost for one treatment course were calculated using the default settings of the automated Microsoft Excel workbook developed by WHO and HAI [6], with a single exception: the Excel workbook allowed to differentiate between district hospitals and health centers only in case of public facilities, not in case of faith-based health facilities. Since in Rwanda, the same rules about medicine availability apply to public and faith-based facilities, the availability of misoprostol, omeprazole, ceftriaxone and simvastatin (expected to be available at hospital but not at health center level) in faith-based facilities was calculated manually, only considering their availability at the five faith-based district hospitals. Further analysis and generation of tables and figures were also done using Microsoft Excel.

#### Measurement of availability

Following WHO and HAI methodology [6], medicines availability was calculated by sector as the percentage of facilities which had at least one unit of each medicine in stock at the time of the visit. Mean availability, across the survey medicines, by sector was also calculated.

As shown in Table 1, according to the REML misoprostol, omeprazole, ceftriaxone and simvastatin are expected to be available at hospitals but not at health center level. This rule applies to both public and faith-based health facilities. Accordingly, for these four medicines availability in the public and faith-based sectors was calculated considering only their availability in hospitals (3 public hospitals, 5 faith-based hospitals).

#### Measurement of prices

Price data were only collected if the medicine was physically in stock at the time of the visit.

International reference prices were used as external benchmarks to assess Rwandan prices. The source of the reference prices was the 2015 Management Science for Health International Medical Product Price Guide (MSH-IMPPG), which was the most recent version available [14]. These prices are medians of procurement prices offered by for-profit and not-for-profit suppliers to governments or large not-for-profit “Non-Governmental Organizations” for generics, and are therefore relatively low priced and represent efficient bulk procurement prices. The reference prices, given in US$, were converted to local currency (1 US$ = 902.1530 Rwandan francs). Median values of the observed local medicine prices were compared to the international procurement prices published in the MSH-IMPPG, resulting in the Median Price Ratios (MPRs). Prices were expressed in Rwandan francs (Rwf) and as MPR:

$$Medicine\;Price\;Ratio\;(MPR)=\frac{Median\;unit\;price\;in\;Rwf}{International\;reference\;unit\;price\;in\;Rwf}$$

MPRs were calculated only if at least four prices for the respective medicine were available from a health sector. For example, in the public sector no MPR for misoprostol was calculated as only two prices were obtained for the generic and also the originator brand. In the private sector, no MPR for tranexamic acid (generic) was calculated because there was only a single price obtained.

#### Assessing treatment affordability

Following the WHO and HAI methodology [6], for each medicine affordability was assessed as the number of day’s wages needed by the lowest paid unskilled government worker to purchase a course of treatment based on standard treatment regimens. Treatment courses requiring more than 1 day’s wages are considered unaffordable by WHO and HAI. Based on the information given by Minimum-Wage.org [15], one day’s wage of the lowest paid unskilled government worker in Rwanda was 1,000 Rwf. The number of tablets/units of each medicine required for a course of treatment is shown in Table 1. For medicines from the WHO and HAI global core list, the number of units for a course of treatment are defined in the WHO and HAI manual [6]. For the five medicines on the supplementary list, the “Rwanda Gynecology and Obstetrics Guidelines” and the WHO Model Formulary 2008 were used to define the daily dose and treatment duration for each medicine [16,17].

## Results

### Medicine availability

**Fig 1:**
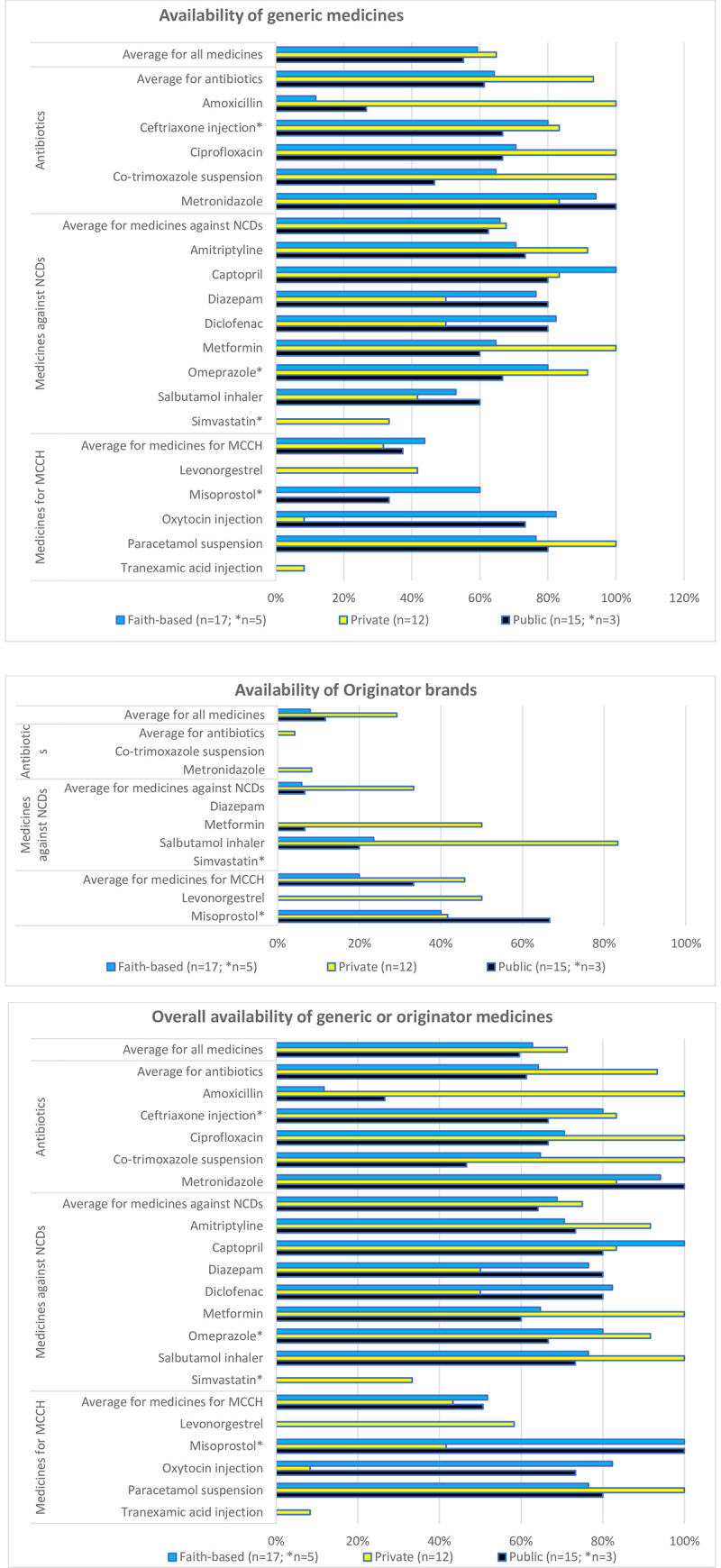
Availability of the investigated medicines in public health facilities, faith-based health facilities, and private pharmacies. a) Availability of generic medicines. b) Availability of originator brands. c) Overall availability of the medicines (generic or originator). * Medicines allowed at hospital level only.

Availability of generic medicines (Fig 1A) was, on average, 64.8% in the private pharmacies, 59.3% in the faith-based health facilities, and 55.2% in the public sector.

Of the eight originator brands (Fig 1B), three were not available in any of the investigated facilities. Mean availability of these eight originator brands was 29.2% in the private pharmacies, 11.7% in the public sector, and 7.9% in the faith-based health facilities.

For salbutamol inhaler and misoprostol tablets, several public and faith-based health facilities were found to stock only the originator brand but not a generic medicine. The prices of these two originator brand medicines were not very different from the prices of the respective generic medicines.

Fig 1C gives the availability of generic and originator combined for each medicine by sector. Availability of all investigated medicines was highest in the private sector (71.3%) and slightly lower in the faith-based (62.8%) and public (59.6%) sectors. For the investigated antibiotics, this combined availability was, on average, 93.3% in the private sector, and 64.2% and 61.3% in the faith-based and the public sector, respectively. Notably, amoxicillin 500mg tablets/capsules showed low availability in public and faith-based facilities, and were not in stock even in several of the hospitals. However, other strengths (such as 250mg tablets/capsules) may have been in stock.

Availability of the medicines used to treat NCDs in private, faith-based and public facilities was 75.0%, 68.8% and 64.2%, respectively, therefore not very different from the results for antibiotics. Simvastatin was unavailable in all the sampled public and faith-based hospitals, despite its inclusion into the REML.

Oxytocin, a life-saving medicine against post-partum hemorrhage, showed availabilities in public (73.3%) and faith-based health facilities (82.4%) close to the 80% target set by the WHO [4,18], while private pharmacies (8.3%) rarely stocked oxytocin injection. Misoprostol is used for induction of labor, against post-partum hemorrhage and for abortions, as well as for the prevention and treatment of stomach ulcers. According to the REML, it should be available in hospitals, and indeed was found in stock in all hospitals sampled in this study. It was also found in one public health center, one faith-based health center, and in five of the twelve private pharmacies. Tranexamic acid is not included in REML, and correspondingly was found only in a single facility, which was a private pharmacy. The contraceptive levonorgestrel is included in REML and should be available both in health centers and in hospitals. In this study, however, it was only found in private pharmacies.

### Medicine prices

**Fig 2:**
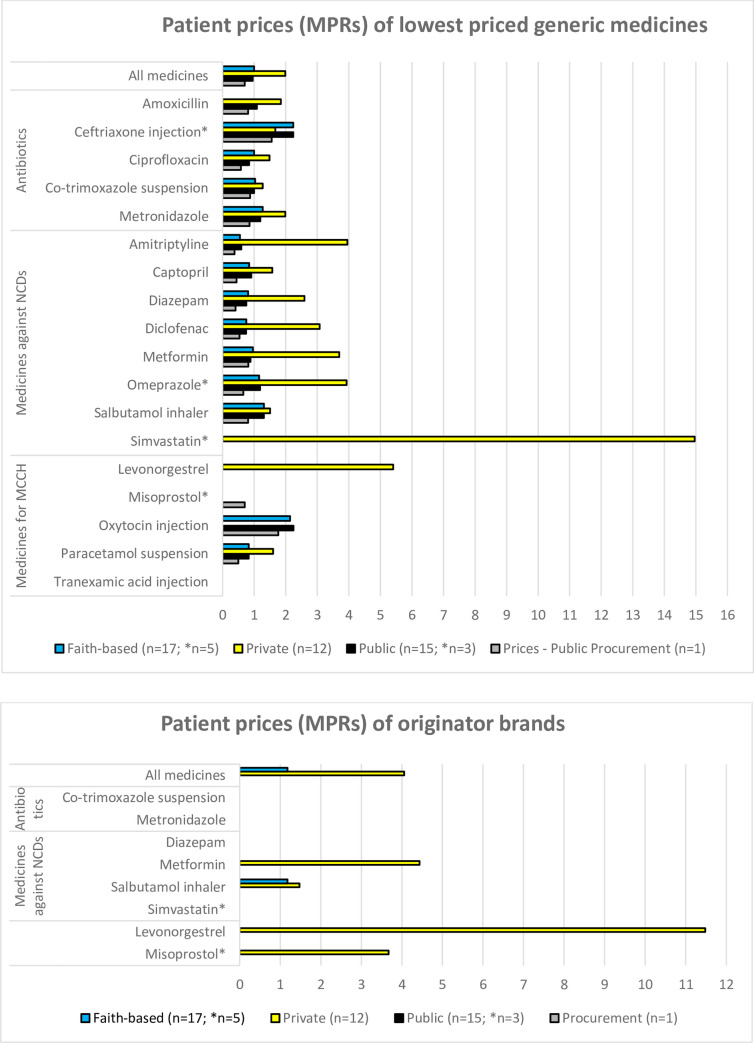
Median price ratios of the investigated medicines in public health facilities, faith-based health facilities, private pharmacies, and in public procurement. a) Lowest-priced generic medicines; b) Originator brands.

For lowest priced generics in faith-based and public health facilities (Fig 2A), median MPRs were 1.0 in both cases. In private pharmacies, the median MPR was twice as high (MPR = 1.99).

Overall, government procurement was efficient for lowest priced generics, with a median MPR of 0.7, i.e. prices 30% below the international reference prices. For 16 out of 18 surveyed medicines, the MPR was less than one. The two medicines where the government procurement price exceeded the international reference price were oxytocin injection (MPR 1.76) and ceftriaxone injection (MPR 1.55).

#### Patient prices of individual medicines

In public and faith-based health facilities, MPRs for lowest priced generics used to treat NCDs were slightly lower than those for antibiotics. Conversely, in private pharmacies the MPRs for generic medicines used to treat NCDs were higher than those for antibiotics (Fig 2A). Levonorgestrel and simvastatin generics were only found in private pharmacies, not in public or faith-based health facilities. This lack of competition translated into remarkably high prices in the private sector, reaching an MPR of 15 for lowest priced generics of simvastatin.

As mentioned above, originator brands were rarely found in public and faith-based health facilities, and even in private pharmacies, the required number of four prices for the calculation of an MPR was only reached for four medicines (Fig 2B). The overall MPR for those four brands in the private sector was 4.05, ranging from 1.48 for salbutamol to 11.48 for levonorgestrel.

A paired analysis of patient prices for lowest priced generics and originator brands in each sector was not possible due to the few originator brands where an MPR was calculated.

For salbutamol inhalers in the private and faith-based sectors (Fig 2A and 2B) and for misoprostol tablets in the faith-based sector (S2 Table), the median prices of the originator brands were not dissimilar from the median prices of the respective lowest priced generics (note: the misoprostol data was based on few data points).

S2 Table gives the median prices in local currency.

#### Cross sector price comparisons

Based on a paired analyses of lowest priced generics:

- patient prices in the public health facilities were 30% higher than the government procurement prices (14 medicines);
- patient prices in private pharmacies were 103% higher than in public facilities (13 medicines);
- Patient prices in faith-based facilities were 10% higher than in public sector facilities (13 medicines); and
- Patient prices in faith-based facilities were 47% lower than in private pharmacies (12 medicines)

There was insufficient data to do across sector paired analyses for originator brands.

### Medicines affordability

As explained in the Methods section, affordability was expressed as the number of day’s wages of the lowest paid unskilled government worker required to purchase a course of treatment based on standard treatment regimens [6]. For chronic diseases, the cost for 30 days of treatment were used in this calculation.

**Fig 3:**
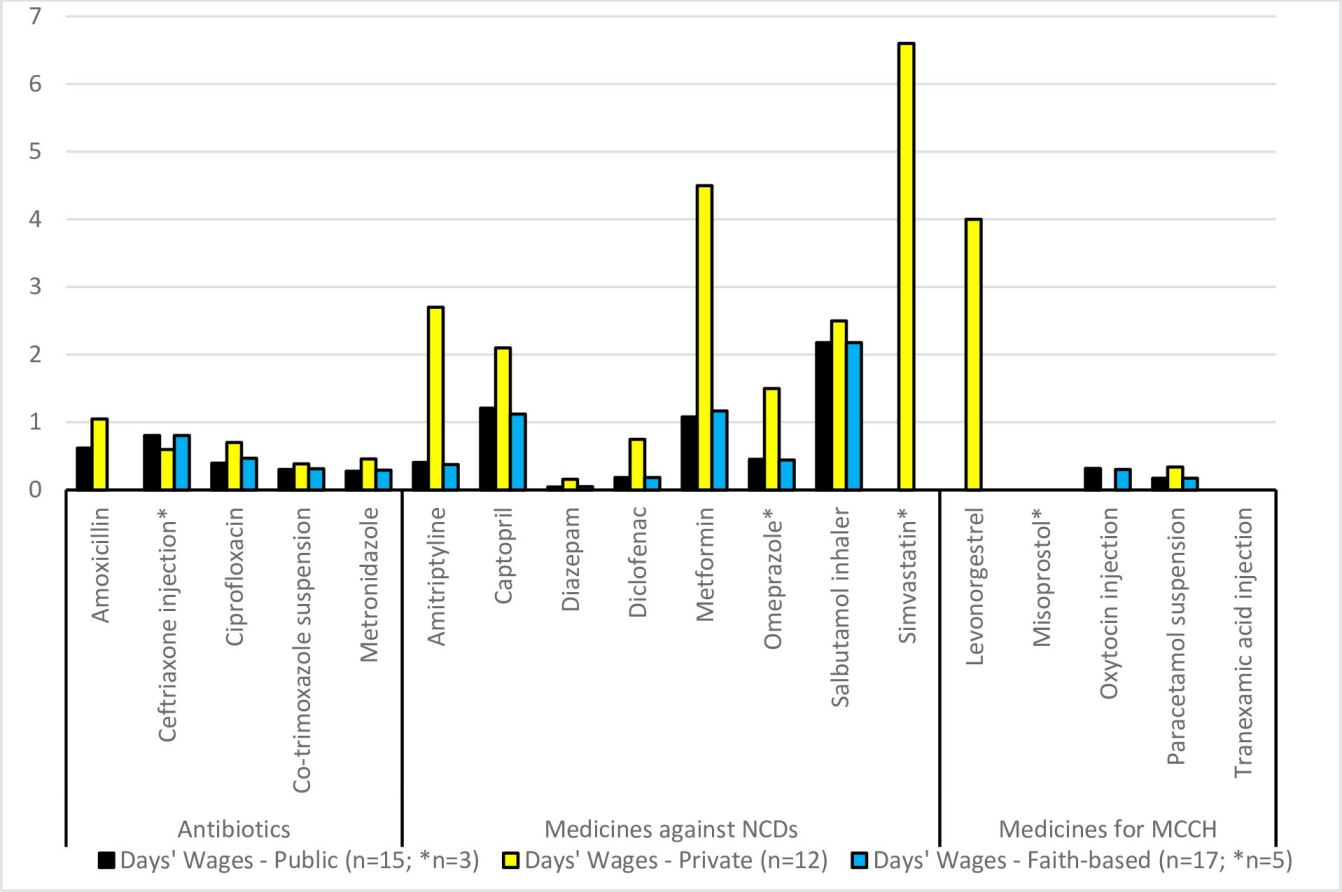
Number of day’s wages required to purchase a course of treatment of the lowest-priced generic medicine.

According to WHO/HAI, treatments are considered unaffordable if they require more than one day’s wage. As shown in Fig 3, antibiotic treatments with lowest priced generics were affordable, even when purchased from private pharmacies. It should be noted that for ceftriaxone, the treatment regimen was for a single dose of 1g. If the “WHO Model Prescribing Information” for pneumonia were followed [19], a course of treatment would consist of 1-2g per day for 7 days, therefore treatment costs of ceftriaxone would result 7–14 times higher than shown in Fig 3.

Of the eight medicines for chronic diseases, six required more than one day’s wage if lowest priced generics were purchased in a private pharmacy, with simvastatin being the least affordable treatment (6.6 days wages for 30 tablets). Oxytocin, the most important life-saving medicine for post-partum hemorrhage, was found affordable at 0.3 days wages.

All four treatments with originator brands purchased from private pharmacies were found to be unaffordable, ranging from 2.4–8.5 days’ wages. S3 Table gives the treatments, their median patient price in Rwf, and the number of day’s wages required to purchase the treatment.

In Rwanda, 92% of the population are covered by the nation’s health insurance schemes [20]. By far the largest part of the population, including the people in the informal sector of rural Rwanda, is covered by the Community Based Health Insurance (CBHI), also commonly known as Mutuelle de Santé. Public servants and employees of the formal sector are covered by the Medical Insurance Scheme (MIS) provided by the Rwanda Social Security Board (RSSB), and members of the armed forces are insured by the Medical Military Insurance (MMI) [21]. The CBHI covers (at least) 90% of the total cost of health care services, and this includes 90% of the cost for essential medicines in the public and faith-based sectors [22,23].

This provision renders all treatments with lowest-price generic medicines in the public and faith-based sectors affordable. However, the problem is that the availability of medicines in the public and faith-based sectors was often much lower than the 80% availability target set by WHO for NCDs medicines. Therefore, patients have to revert to the private sector to obtain these medicines, and since CBHI does not cover the cost of medicines from the private sector, patients have to pay 100% of the costs, which renders many treatments for chronic diseases unaffordable (Fig 3).

## Discussion

Access to quality-assured essential medicines has been defined as a priority by WHO in the struggle to meet the Sustainable Development Goal No. 3 of the United Nations [1,2]. The results of the present study on the availability, prices and affordability of important medicines is a contribution to assessing progress towards this goal in Rwanda.

Median public procurement prices for 16 out of 18 generic medicines were lower than the international supplier price given in the 2015 MSH-IMPPG [14]. This shows efficient procurement procedures in the Rwandan public sector. This also translated into remarkably low patient prices in the public and faith-based sectors, due to proper regulation of the prices in the Rwandan public health system [21]. As usual, patient prices in the private sector were clearly higher; for example, the patient price for amitriptyline tablets 25mg in private pharmacies was more than 6-times its government procurement price. Obviously, higher prices in the private sector can be justified by price components in the supply chain such as salaries, rent for premises, transportation means when importing medicines, etc. Nearly all previous studies in LMICs found that prices were higher in the private sector than in the public and the faith-based sectors [24]. An exception is a study from Malawi which found prices of antibiotics and antimalarials in the faith-based sector to be higher than in the private sector [25].

According to WHO, availability of essential medicines for NCDs should be at least 80% [4,18]. However, in the present study the availability of most of the essential medicines in public and faith-based health facilities in Rwanda was found to fall short of this WHO benchmark. This situation is unfortunately quite common in many LMICs. E.g. an authoritative review published in “The Lancet” reported that the average availability of generic medicines in public health facilities of 36 LMICs ranged from 29.4% to 54.4% [24]. In Rwanda, the availability of the 18 essential medicines investigated in this study was mostly higher than in the 36 countries investigated in that review, but lower than e.g. the availability of 20 essential medicines in clinics and health centers in Ghana in 2013 [26]. On the other hand, the availability of generic medicines in the public and private sectors of Shaanxi Province, Western China [27] was lower than found in the present study for Rwanda.

The results on medicine affordability obtained in this study have to be placed in the context of the Rwandan health system and the organization of its pharmaceutical sector. The Medical Production, Procurement and Distribution Division (MPDD) of the Rwanda Biomedical Center is the most important supplier of medicines to all public institutions. Faith-based health facilities obtain their medicines also from MPDD, or from the Bureau des Formations Médicales Agréées du Rwanda (BUFMAR), while private pharmacies purchase from private wholesalers.

The medicine pricing and reimbursement system of Rwanda has been described in detail in a study by WHO in 2016 [21]. As mentioned above, most of the population of Rwanda is covered by the Community Based Health Insurance (CBHI) scheme. CBHI is managed by the Rwanda Social Security Board (RSSB), which is under supervision of the Ministry of Finance and Economic Planning. CBHI covers costs for health services and medicines in public or faith-based health facilities in Rwanda.

The patient prices of medicines in the public and faith-based health facilities are regulated by ministerial instruction No. 20/1658/PTF/2007 of 15 June 2007, allowing a maximum mark-up of 20% over the procurement prices [21]. No national medicine pricing policy exists for the private sector; for patients covered by the Medical Insurance Scheme of RSSB, this insurance allows a maximum profit margin of 40% over the private wholesalers’ prices [21].

For patients covered by the Community Based Health Insurance (CBHI), the annual insurance fees per person as well as their co-payments for health services and medicines depend on the socio-economic class of the family, known as “Ubudehe” category. Persons in categories 1&2 (24% of the total population) do not pay any fees or co-payments for health services. Persones in the higher categories 3–6 pay annual insurance fees as well as a flat fee of 200 RWF at the health center level, and 10% of each bill (consultation, diagnosis and medicines) at the hospital level as co-payments. At the end of the month, each partner health facility submits an invoice to the Rwanda’s CBHI for payment of the uncovered fees, for treated patients during the month [22]. The historical development of this community-based health insurance in Rwanda has been described in a study by the University of Rwanda and Management Sciences for Health [23].

Considering the excellent health insurance coverage in Rwanda [21], treatments with the medicines investigated in this study were affordable in the public and faith-based sectors, setting a positive example for other LMICs in the strive for UHC. However, in the private sector treatments for chronic diseases, such as amitriptyline, captopril, metformin, salbutamol inhaler and simvastatin, were largely unaffordable. The same situation was found in the private sector of 36 low- and middle-income countries [24]. This is a serious financial problem for the patients who have to take these medicines for long time periods, usually for life.

This study identified a key issue for patients in Rwanda, that is, that the availability of medicines in the public and faith-based sectors was often low, forcing them to buy in the private sector at far higher prices and no CBHI scheme coverage. The reasons for this sub-optimal availability needs to be identified, and actions taken to improve the situation. In addition, systems need to be put into place for regularly monitoring the availability, prices and affordability of key essential medicines in the public, private and faith-based sectors of Rwanda.

## Limitations of the study

The study included a small range of medicines, few of which had originator brands registered in the country. It was originally intended to include also private clinics into this study. However, in Rwanda private clinics are not supposed to store medicines except for few emergency medications, therefore private clinics had to be excluded from the study. Medicine prices in Rwanda were collected in 2019, but the latest available version of the MSH-IMPPG was for 2015. In addition, this survey was conducted at one point in time only, so it does not take into account availability and prices changes over time.

## Supporting information

S1 Table Availability of surveyed medicines. (PDF)

S2 Table Medicines prices expressed as Median Price Ratios (MPRs). (PDF)

S3 Table Medicines affordability expresed as amount of local currency (Rwf) and number of day’s wages required for one course of treament. (PDF)

## References

[1] United Nations Economic and Social Council. Special edition: Progress towards the Sustainable Development Goal—Report of the Secretary-General. 2019. Available from: https://sustainabledevelopment.un.org/content/documents/22700E_2019_XXXX_Report_of_the_SG_on_the_progress_towards_the_SDGs_Special_Edition.pdf

[2] Burrone E, Gotham D, Gray A, de Joncheere K, Magrini N, Martei YM, et al Patent pooling to increase access to essential medicines. Bulletin of the World Health Organization. 2019;97(8):575–7. 10.2471/BLT.18.229179 31384076 PMC6653814

[3] Wirtz VJ, Hogerzeil H V., Gray AL, Bigdeli M, De Joncheere CP, Ewen MA, et al Essential medicines for universal health coverage. Lancet. 2017; 389(10067):403–476. 10.1016/S0140-6736(16)31599-9 27832874 PMC7159295

[4] World Health Organization. Sixty-sixth World Health Assembly. Resolutions and Decisions. Annexes. Geneva; 2013. Available from: http://apps.who.int/gb/ebwha/pdf_files/WHA66-REC1/WHA66_2013_REC1_complete.pdf

[5] UN Commission on Life-Saving Commodities for Women and Children. Every woman Every child. Commissioners’ Report—September 2012. New York; 2012. Available from: www.unfpa.org/sites/default/files/pub-pdf/Final UN Commission Report_14sept2012.pdf

[6] World Health Organization, Health Action International. Measuring medicine prices, availability, affordability and price components. 2nd ed Falvey M, editor. Health Action International Geneva; 2016.

[7] Health Action International. Database of medicine prices, availability, affordability and price components. Available from: https://haiweb.org/what-we-do/price-availability-affordability/price-availability-data/

[8] Nditunze L, Makuza S, Amoroso CL, Odhiambo J, Ntakirutimana E, Cedro L, et al Assessment of essential medicines stock-outs at health centers in Burera district in northern Rwanda. Rwanda J. Ser. F: Med. Health Sci. 2015 10 8;2(1):85.

[9] Ministry of Health—Rwanda. National list of essential medicines for paediatrics. 1st ed. 2015. Available from: www.moh.gov.rw/fileadmin/templates/Docs/NEML_For_Paediatrics-_1st_Edition-2015.pdf

[10] Ministry of Health—Rwanda. National list of essential medicines for adults. 6th ed. Kigali; 2015. 25 p. Available from: www.medbox.org/essential-medicines-lists/national-list-of-essential-medicines-for-adults-rwanda-6th-edition/preview?

[11] WHO. Updated WHO recommendation on tranexamic acid for the treatment of postpartum haemorrhage. 2017. Available from: https://apps.who.int/iris/bitstream/handle/10665/259379/WHO-RHR-17.21-eng.pdf?sequence=1 29630190

[12] Ministry of Health—Rwanda. List of authorized medicines August 2017. Kigali; 2017. Available from: www.moh.gov.rw/fileadmin/user_upload/AUTHORIZED_MEDICINES_AUGUST_2017.pdf

[13] Ministry of Health—Rwanda. Private health facilities in Rwanda health service packages. January 20. Kigali; 2017. http://moh.gov.rw/fileadmin/templates/Norms/Private_Health__Facilities_service_packages__in_Rwanda.pdf

[14] MSH (Management Sciences for Health). International Medical Products Price Guide. 2015th ed Frye JE, editor. Medford, MA 02155 USA: Mass.: MSH; 2016 Available from: http://apps.who.int/medicinedocs/documents/s23203en/s23203en.pdf

[15] Minimum-Wage.org. Denmark Minimum Wage, Labor Law, and Employment Data Sheet Denmark Minimum Wage Rate 2018. Available from: www.minimum-wage.org/international/denmark

[16] Ministry of Health—Rwanda. Gynecology and obstetrics—Clinical protolols and treatment guidelines. September. Kigali; 2012. 27 p. Available from: www.moh.gov.rw/fileadmin/templates/Clinical/OBS_Gyn_last-version.pdf

[17] World Health Organization WHO Model Formulary 2008. Stuart MC, Kouimtzi M, Hill SR, editors. Geneva: WHO Press; 2009 Available from: https://apps.who.int/medicinedocs/documents/s16879e/s16879e.pdf

[18] World Health Organization Global action plan for the prevention and control of noncommunicable diseases 2013–2020. Geneva: WHO Press; 2013 55 p. Available from: www.who.int/about/licensing/copyright_form/en/index.html

[19] World Health Organization. WHO model prescribing information: Drugs used in bacterial infections: lower respiratory tract infections: pneumonia. Available from: https://apps.who.int/medicinedocs/en/d/Js5406e/4.3.html#Js5406e.4.3

[20] Pacific Prime International. Rwanda health insurance. Available from: https://www.pacificprime.com/country/africa/rwanda-health-insurance/

[21] World Health Organisation. Assessment of medicine pricing and reimbursement systems in health insurance schemes in selected african countries. World Health Organisation 2016 Available from: http://apps.who.int/iris/bitstream/handle/10665/246416/9789290233145-eng.pdf?sequence = 1

[22] Republic of Rwanda—Ministry of Health. Ministry of Health annual report 2012–2013. Kigali; 2013. Available from: http://moh.gov.rw/fileadmin/templates/Press_release/MoH_Annual_Report_July_2012-June_2013.pdf

[23] Kalisa I, Musange SF, Collins D, Saya U, Kunda T, Parfait U, et al The development of community-based health insurance in Rwanda: Experiences and lessons. University of Rwanda College of Medicine and Health Sciences—School of Public Health, Kigali, Rwanda and Management Sciences for Health, Medford, MA, USA 2015 Available from: https://www.msh.org/sites/msh.org/files/the_development_of_cbhi_in_rwanda_experiences_and_lessons.pdf

[24] Cameron A, Ewen M, Ross-Degnan D, Ball D, Laing R. Medicine prices, availability, and affordability in 36 developing and middle-income countries: a secondary analysis. Lancet. 2009;373(9659):240–9. 10.1016/S0140-6736(08)61762-6 19042012

[25] Khuluza F, Heide L. Availability and affordability of antimalarial and antibiotic medicines in Malawi. PLoS One. 2017;12(4):1–16. 10.1371/journal.pone.0175399 PMC5395150 28419126

[26] Nyanwura EM, Esena RK. Essential medicines availability and affordability: a case study of the top ten registered diseases in Builsa district of Ghana. Int J Sci Technol Res. 2013;2(8):208–19.

[27] Fang Y, Jiang M. Medicine prices, availability and affordability in Shaanxi Province, Western China. 2012. Available from: http://apps.who.int/medicinedocs/documents/s20143en/s20143en.pdf

