# Supplementary material for: Prices, availability and affordability of medicines in Rwanda
Source: PLoS One. 2020 Aug 3;15(8):e0236411. doi: 10.1371/journal.pone.0236411 (PMC7398547; doi:10.1371/journal.pone.0236411)
Supplement: S1 Table — (PDF) [file pone.0236411.s001.pdf]

S1 Table: Availability of surveyed medicines

Class of medicines/ indication	Surveyed medicines		Originator brand			Lowest price generic			Originator and lowest price generic combined		
	**Medicine Name**	**Medicine list**	**Public (n=15)**	**Private (n=12)**	**Faith-based (n=17)**	**Public (n=15)**	**Private (n=12)**	**Faith-based (n=17)**	**Public (n=15)**	**Private (n=12)**	**Faith-based (n=17)**
**Medicines for MCCH**	Tranexamic acid injection	Supplementary				0.0%	8.3%	0.0%	0.0%	8.3%	0.0%
	Paracetamol suspension	Global				80.0%	100.0%	76.5%	80.0%	100.0%	76.5%
	Oxytocin injection	Supplementary				73.3%	8.3%	82.4%	73.3%	8.3%	82.4%
	Misoprostol	Supplementary	66.7%	41.7%	40.0%	33.3%	0.0%	60.0%	100.0%	41.7%	100.0%
	Levonorgestrel	Supplementary	0.0%	50.0%	0.0%	0.0%	41.7%	0.0%	0.0%	58.3%	0.0%
	**Average for medicines for MCCH**		33.3%	45.8%	20.0%	37.3%	31.7%	43.8%	50.7%	43.3%	51.8%
**Medicines against NCDs**	Simvastatin	Global	0.0%	0.0%	0.0%	0.0%	33.3%	0.0%	0.0%	33.3%	0.0%
	Salbutamol inhaler	Global	20.0%	83.3%	23.5%	60.0%	41.7%	52.9%	73.3%	100.0%	76.5%
	Omeprazole	Global				66.7%	91.7%	80.0%	66.7%	91.7%	80.0%
	Metformin	Global	6.7%	50.0%	0.0%	60.0%	100.0%	64.7%	60.0%	100.0%	64.7%
	Diclofenac	Global				80.0%	50.0%	82.4%	80.0%	50.0%	82.4%
	Diazepam	Global	0.0%	0.0%	0.0%	80.0%	50.0%	76.5%	80.0%	50.0%	76.5%
	Captopril	Global				80.0%	83.3%	100.0%	80.0%	83.3%	100.0%
	Amitriptyline	Global				73.3%	91.7%	70.6%	73.3%	91.7%	70.6%
	**Average for medicines against NCDs**		6.7%	33.3%	5.9%	62.5%	67.7%	65.9%	64.2%	75.0%	68.8%
**Antibiotics**	Metronidazole	Supplementary	0.0%	8.3%	0.0%	100.0%	83.3%	94.1%	100.0%	83.3%	94.1%
	Co-trimoxazole suspension	Global	0.0%	0.0%	0.0%	46.7%	100.0%	64.7%	46.7%	100.0%	64.7%
	Ciprofloxacin	Global				66.7%	100.0%	70.6%	66.7%	100.0%	70.6%

	Ceftriaxone injection	Global				66.7%	83.3%	80.0%	66.7%	83.3%	80.0%
	Amoxicillin	Global				26.7%	100.0%	11.8%	26.7%	100.0%	11.8%
	**Average for antibiotics**		0.0%	4.2%	0.0%	61.3%	93.3%	64.2%	61.3%	93.3%	64.2%
	**Average for all medicines**		11.7%	29.2%	7.9%	55.2%	64.8%	59.3%	59.6%	71.3%	62.8%
