# Supplementary material for: Prices, availability and affordability of medicines in Rwanda
Source: PLoS One. 2020 Aug 3;15(8):e0236411. doi: 10.1371/journal.pone.0236411 (PMC7398547; doi:10.1371/journal.pone.0236411)
Supplement: S2 Table — (PDF) [file pone.0236411.s002.pdf]

S2 Table: Medicines prices expressed as Median Price Ratios (MPRs)

Class of medicine	Medicine name	Originator brand				Lowest-price generic			
		Govt. Procurement	Patient MPR			Govt. Procurement MPR	Patient MPR		
			Public (n=15)	Private (n=12)	Faith-based (n=17)		Public (n=15)	Private (n=12)	Faith-based (n=17)
Antibiotics	Amoxicillin capsule 500mg					0.81	1.09	1.85	
	Ceftriaxone injection 1g					1.55	2.24	1.67	2.24
	Ciprofloxacin tablet 500mg					0.58	0.84	1.49	1.00
	Co-trimoxazole suspension 8+40mg/ml					0.88	1.00	1.27	1.03
	Metronidazole tablet 250mg					0.85	1.19	1.99	1.27
Medicines against NCDs	Amitriptyline tablet 25mg					0.38	0.59	3.96	0.55
	Captopril tablet 25mg					0.44	0.91	1.58	0.84
	Diazepam tablet 5mg					0.41	0.75	2.60	0.81
	Diclofenac tablet 50mg					0.54	0.75	3.08	0.75
	Metformin tablet 500mg			4.43		0.81	0.89	3.69	0.96
	Omeprazole capsule 20mg					0.66	1.19	3.93	1.16
	Salbutamol inhaler 100mcg/dose			1.48	1.18	0.81	1.31	1.51	1.31
	Simvastatin tablet 20mg							14.96	
Medicines for MCH	Levonorgestrel tablet 1.5mg			11.48				5.40	
	Misoprostol tablet 200mcg			3.67		0.70			
	Oxytocin injection 10IU/ml					1.76	2.25		2.14
	Paracetamol suspension 24mg/ml					0.50	0.83	1.60	0.83
	Tranexamic acid injection 100mg/ml								
Median	All medicines			4.05	1.18	0.70	0.96	1.99	1.00
