# Supplementary material for: Prices, availability and affordability of medicines in Rwanda
Source: PLoS One. 2020 Aug 3;15(8):e0236411. doi: 10.1371/journal.pone.0236411 (PMC7398547; doi:10.1371/journal.pone.0236411)
Supplement: S3 Table — (PDF) [file pone.0236411.s003.pdf]

S3 Table: Medicines affordability expresed as amount of local currency (Rwf) and number of day's wages required for one course of treament

Surveyed medicines		Originator brand						Lowest-price generic					
		Public (n=15)		Private (n=12)		Faith-based (n=17)		Public (n=15)		Private (n=12)		Faith-based (n=17)	
Class of medicines	Treatment	Median Treatment Price (Rwf)	Days' Wages	Median Treatment Price (Rwf)	Days' Wages	Median Treatment Price (Rwf)	Days' Wages	Median Treatment Price (Rwf)	Days' Wages	Median Treatment Price (Rwf)	Days' Wages	Median Treatment Price (Rwf)	Days' Wages
Antibiotics	Amoxicillin capsule 500mg x21							617.4	0.6	1050.0	1.1		
	Ceftriaxone injection 1g x1							803.5	0.8	600.0	0.6	803.8	0.8
	Ciprofloxacin tablet 500mg x14							394.8	0.4	700.0	0.7	470.4	0.5
	Co-trimoxazole suspension 8+40mg/ml 70ml							303.8	0.3	385.0	0.4	312.2	0.3
	Metronidazole tablet 250mg x42							275.9	0.3	459.9	0.5	294.0	0.3
Medicines against NCDs	Amitriptyline tablet 25mg x90							405.0	0.4	2700.0	2.7	375.4	0.4
	Captopril tablet 25mg x60							1210.5	1.2	2100.0	2.1	1122.0	1.1
	Diazepam tablet 5mg x7							45.5	<0.1	157.5	0.2	49.0	<0.1
	Diclofenac tablet 50mg x60							181.5	0.2	750.0	0.8	183.0	0.2
	Metformin tablet 500mg x90			5400.0	5.4			1080.0	1.1	4500.0	4.5	1170.0	1.2
	Omeprazole capsule 20mg x30							452.9	0.5	1500.0	1.5	440.9	0.4
	Salbutamol 100mcg/dose 200 dose inhaler			2450.0	2.5	1954	2.0	2178.0	2.2	2500.0	2.5	2178.0	2.2
	Simvastatin tablet 20mg x30									6600.0	6.6		
Medicines for MCH	Levonorgestrel tablet 1.5mg x1			8500.0	8.5					4000.0	4.0		

	Misoprostol tablet 200mcg x4			2400.0	2.4								
	Oxytocin injection 10IU/ml x1ml							316.0	0.3			300.9	0.3
	Paracetamol suspension 24mg/ml x45ml							175.0	0.2	337.5	0.3	174.2	0.2
	Tranexamic acid injection 100mg/ml												
